# First-episode psychosis and substance use in Nelson Mandela Bay: Findings from an acute mental health unit

**DOI:** 10.4102/sajpsychiatry.v25i0.1372

**Published:** 2019-10-24

**Authors:** Yanga Thungana, Zukiswa Zingela, Stephan van Wyk

**Affiliations:** 1 Department of Psychiatry and Behavioural Sciences, Walter Sisulu University, Mthatha, South Africa; 2 Acute Mental Health Care Unit, Dora Nginza Hospital, Bethelsdorp, South Africa; 3 Nelson Mandela Academic Hospital, Mthatha, South Africa

**Keywords:** substance use, first-episode psychosis, dual diagnosis, cannabis use, polysubstance use

## Abstract

**Background:**

Use of psychoactive substances is a common finding in studies on first-episode psychosis (FEP), and this has prognostic implications. We know very little about psychoactive substance use (SU) among patients with FEP in the Eastern Cape province (EC) of South Africa (SA).

**Aim:**

The study seeks to determine SU prevalence and associated features among inpatients with non-affective FEP in an acute mental health unit (MHU) in Nelson Mandela Bay, EC.

**Setting:**

Researchers conducted a retrospective clinical file review of a 12-month admission cohort of patients with FEP, without a concurrent mood episode, to the Dora Nginza Hospital MHU. Information collected included SU history, psychiatric diagnoses, and demographics. Data were then subjected to statistical analysis.

**Methods:**

Researchers conducted a retrospective clinical file review of a 12-month admission cohort of patients with FEP, without a concurrent mood episode, to the Dora Nginza Hospital MHU. Information collected included SU history, psychiatric diagnoses and demographics. Data were then subjected to statistical analysis.

**Results:**

A total of 117 patients (86 [73.5%] males; 31 [26.5%] females) aged 18–60 years (mean 29 years) met the inclusion criteria. After controlling for missing information, 95 of 117 (81.2%) patients had a history of active or previous SU, 82 of 90 (91.1%) were single and 61 of 92 (66.3%) were unemployed. A significant association was found between SU and unemployment (*p* < 0.001), as well as male sex (*p* < 0.001). The most common substances used were cannabis (59.8%), followed by alcohol (57.3%) and stimulants (46.4%).

**Conclusion:**

In keeping with national and international literature, the results of this study showed a high prevalence of substance use in South African patients with first-episode psychosis. The high prevalence of lifetime substance use in this cohort compared to previous studies in South Africa requires further investigation and highlights the urgent need for dual diagnosis services in the Eastern Cape province.

## Introduction

The use of psychoactive substances is a common finding among patients with first-episode psychosis (FEP).^1,2,3,4^ Several studies report that psychoactive substance use (SU) has negative prognostic implications for short- and long-term outcomes of FEP.^5,6,7,8,9^ Other studies report that FEP associated with SU is more likely to be acute in onset with a shorter period of untreated psychosis, although patients are more likely to be admitted involuntarily at first psychiatric contact.^7,10,11^ The reported close relationship between SU and emergent psychosis suggests a possible causal link, which has highlighted the importance of early intervention for SU as a way of improving outcomes in FEP.^12,13^

The prevalence of SU among patients presenting with FEP ranges from 30% to 75% across studies and countries.^1,9,14,15,16^ The wide variation is possibly because of a combination of methodological inconsistencies, as well as cultural and environmental differences between countries, especially regarding the availability of illicit substances.^16^ The types of substances used by people with FEP vary across studies, but cannabis and alcohol tend to be the most frequently reported.^2,15,17,18^

In South Africa, the prevalence of alcohol and other drug use disorders is high, with males having higher rates than females, but the gender gap has been narrowing over the last decade.^19,20,21^ Alcohol, tobacco and illicit drugs – among them cannabis and methamphetamine – are the most commonly used substances, with a varying level of use between men and women.^22,23^ Studies that were conducted in Western Cape and KwaZulu-Natal provinces have documented high co-occurrence of substance use in individuals with mental disorders.^24,25,26,27^ The findings from other provinces are not easily generalisable to the Eastern Cape province as the samples differ in ethnicity and socio-economics.

Despite the strong association between substance use and FEP, the researchers could not find any published study on the topic specific to the Eastern Cape (EC) province of South Africa (SA).

## Study aims

The primary aim was to determine the prevalence of SU among inpatients with non-affective FEP on an acute mental health unit (MHU) in Nelson Mandela Bay (NMB), EC. Secondary aims were to identify the predominant substances, as well as clinical and demographic associations.

### Study design

The researchers conducted a retrospective, descriptive study of a 12-month cohort of patients admitted for non-affective FEP to a 35-bed acute MHU in Dora Nginza Hospital (DNH), which is a large regional government hospital in NMB.

## Method

### Procedure

The first author identified all patients admitted to the MHU between 01 November 2016 and 31 October 2017 and systematically scrutinised all available clinical files. Selection criteria were applied, namely: (1) admission for a first-in-lifetime psychotic episode, (2) ages between 18 and 60 years and (3) the simultaneous absence of an episode of a DSM-5 bipolar or major depressive disorder. Clinical files from the inclusion group were then subjected to a data extraction exercise.

### Sample

A total of 1481 patients were admitted to the MHU during the 12-month study period. The multidisciplinary team evaluated all patients. For the study, only individuals with non-affective FEP were included. FEP was defined as individuals with psychosis receiving medical or psychological treatment for the first time and affective symptoms not meeting criteria for a mood disorder using DSM-5 ^28^ diagnostic criteria. A total of 271 patients were identified from the admission register to have FEP. Of those, 154 were excluded because of missing case files, not meeting age criteria, having confirmed previous episodes or affective psychosis. The final sample consisted of 117 patients.

### Data collection

To ensure consistency, a single researcher captured all data using an anonymised data sheet. Information gathered from clinical files included SU history, physical and psychiatric diagnoses, education and other relevant demographics. SU was defined as the use of any psychoactive substance, whether by evidence from medical history or drug testing, and irrespective of time frame, quantity or usage pattern. Nicotine, as well as the non-problem use of alcohol, and psychoactive medications (over-the-counter or prescribed) were excluded.

Problem use of alcohol or medication was taken to mean progressive usage escalation, withdrawal symptoms, cravings, deliberate attempts at discontinuation, detrimental effects on health (e.g. alcohol blackouts and delirium tremens) or negative impact on functioning (relationships, work, education and substance-related legal problems). Current SU meant the use of substance within the last 3 months. Both point-of-care urine drug screening tests and laboratory tests were accepted as evidence of current SU. The urine drug test used on the MHU was a 6-drug immunoassay, which tested for cannabis (delta-9 tetrahydrocannabinol), amphetamine (AMP), methamphetamine (MAMP), cocaine, methaqualone and opiates.

### Data analysis

All data were entered into a single database. Descriptive statistics analysis was performed using frequency tables, proportions, means and standard deviations (s.d.). For inferential statistics, cross-tabulation with the chi-square (*X*^2^) or Fisher exact test was used for categorical variables. Stata 13 was used for all the statistical analysis, and Excel 2017 was used for drawing up the graphical representation of the data. The significance level was set at *p* < 0.05.

### Ethical considerations

Consent to conduct the study was obtained from Walter Sisulu University – Human Research Committee, Eastern Cape Department of Health and institutional managers at Dora Nginza Hospital.

## Results

### Demographics

Of the total sample (*N* = 117), the majority were male (*n* = 86, 73.5%), black (*n* = 77, 65.8%), single (*n* = 89, 76.1%) and unemployed (*n* = 64, 54.7%). The racial breakdown of the 117 patients in the analysis was similar to that of the catchment population.^29^ The male to female ratio in the study was 2.8:1 compared to that of the catchment area (1:1.1).^29^ The age range of the 117 participants was between 18 and 60 years, with a mean of 28.88 years (s.d. 10.63). The majority of participants had some form of high school education (*n* = 86, 71.8%), but only a few had a higher level of education (*n* = 11, 9.4%). Most were brought to the hospital by their families, with a large number needing help from the South African Police Services (see Table 1).

**TABLE 1:** Demographics and clinical characteristics of patients with first-episode psychosis (*N* = 117).

Characteristics	Percentage	Number
Male	73.5	86
Female	26.5	31
**Age, years (by category)**		
18–25	48.7	56
26–35	30.8	36
36–60	20.5	24
**Relationship status**		
Single	76.1	89
Has a partner	9.4	11
Divorced	7.7	9
Missing	6.8	8
**Ethnicity**		
Black	65.8	77
Mixed race	25.6	30
White	4.3	5
Indian	2.6	3
Missing	1.7	2
**Education**		
Primary school	8.6	10
Secondary school	71.8	86
Postsecondary school	9.4	11
Missing	10.3	12
**Source of income**		
Employed	33.3	39
State grant	6.0	7
Unemployed	54.7	64
Missing	6.0	7

### Clinical diagnosis

The majority of participants were assessed to have a substance-induced psychotic disorder (*n* = 51, 43.6%), followed by primary psychotic disorders (*n* = 30, 25.6%), psychotic disorder because of a general medical condition (*n* = 24, 20.5%) and a number with an unspecified psychotic disorder (*n* = 12, 10.3%). The most common general medical conditions were HIV infection (58%) and epilepsy (25%).

### Substance use

**FIGURE 1:**
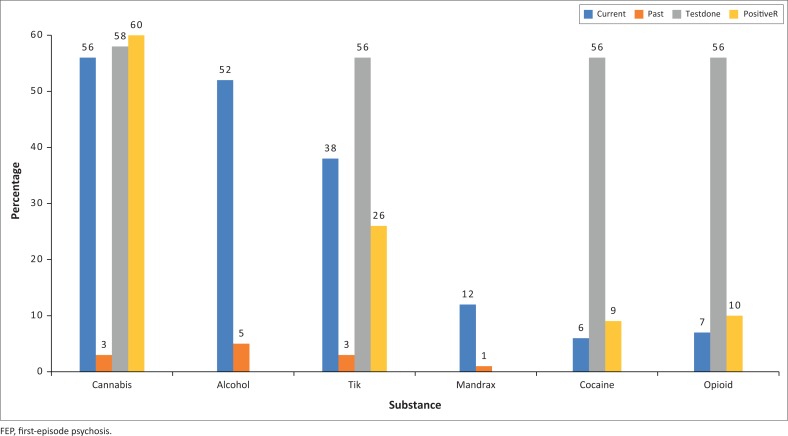
Bar chart showing current and past substance use history, percentage of those tested for substances and positive test results on those who had urine drug testing.

Of the 117 participants, 116 had documented substance history. Of those with documented substance history, 81.9% (*n* = 95) had a lifetime history of substance use. Cannabis was the most commonly used substance (*n* = 70, 59.8%), followed by alcohol (*n* = 67, 57.3%), stimulants (*n* = 52, 46.4%), mandrax (*n* = 14, 12%) and opioid (*n* = 8, 6.8%) (Figure 1). More participants were still actively using substances (cannabis *n* = 66, 94.3%, alcohol *n* = 62, 92.5% and methamphetamines *n* = 42, 93.3%) with only a few who had discontinued substances (5.7% cannabis, 7.5% alcohol and 6.7% methamphetamines).

There were more males (82.1%) with lifetime use of substances than females (17.9%), and this dissimilarity was significant for all substances combined (*p* < 0.001). The difference in sex prevalence was also specifically significant for cannabis (males 90% vs. females 10%; *p* < 0.001), MAMP (males 84.4% vs. females 15.6%; *p* = 0.04) and for alcohol (males 77.6% vs. females 22.4%; *p* < 0.001).

The use of cannabis and MAMP was most prevalent among patients aged 25 years or younger (cannabis mean 25 years; 95 % CI 23.89–26.54; *p* = 0.045 and MAMP mean 24 years; 95% CI 22.5–25.2; *p* = 0.001). In contrast, problem alcohol use was more prevalent above 25 years (mean age 29 years; 95% CI 26.7–31.6; *p* = 0.02). Thirty (31.6%) of the 95 patients with a history of SU had used a single substance, compared to 65 (68.4%) patients who had used more than one substance. Thirty individuals (31.6%) had used two substances, and 35 (36.8%) used three or more substances in their lifetime. The most common combinations were: cannabis–alcohol–MAMP (23.1%), cannabis–alcohol (21.5%) and cannabis–MAMP (18.5%). The average use was 2.2 substances per individual (s.d. 1.1).

Urine drug screening was done in only 68 (58.6) of the 116 patients for whom information on SU was recorded. Of the urine screening tests done, positive tests were for cannabis (60.3%), methamphetamine (26.1%), opiates (10.4%) and cocaine (9.2%). All cocaine and opiate users reported active use, and they all tested positive as well.

Significantly, 55 of the 64 (85.9%) patients who were unemployed had a positive substance use history (*p* < 0.01). There was no significant association between race and substance use (*p* = 0.12) nor between the level of education and substance use (*p* = 0.24).

## Discussion

Our study showed a very high prevalence of substance use (81.9%) in inpatients with FEP who were admitted to the Dora Nginza Mental Health Unit. There were 117 participants with FEP, and 95 (81.2%) of them had used at least one substance group, currently or in the past. This result is higher than published data on substance use rates (30% – 75%) in FEP.^1,14,15,16,30^ This could be because of a combination of factors. Firstly, this could be sampling bias because of the inclusion of patients with both current and lifetime history of substance use. Secondly, this could be because of the perceived rising prevalence of substance use in the area. A study conducted in KwaZulu-Natal in patients with psychotic disorders showed that only 10% had no lifetime history of substance.^27^

In our study, cannabis was the most commonly used substance (59.8%), followed by alcohol (57.3%) and methamphetamines (40.2%). The result is in keeping with many studies which show cannabis and alcohol to be the most commonly used substances in FEP.^1,5,14,26^ The lifetime history of methamphetamines use was higher in our study than previously published data.^15,26,31^ This result could be because of the higher prevalence of substance use in this study sample.

On average, those with cannabis use were younger (25 years) than those with alcohol use (29 years). This is comparable to other studies which show that patients using cannabis presented with a psychotic disorder at an earlier age than those with alcohol use history.^30,32,33^ In our study, 68.4% of those with substance use history had used more than one substance group and most had used three or more substances in a lifetime. Findings of a high prevalence of polysubstance use in FEP have been reported in other studies.^2,34^

Urine drug testing seemed to have been driven by positive substance use history in this study. For example, 59.8% of participants were found to have a documented use of cannabis, and 58% of the study sample had a cannabis urine drug test. It was 60.3% and 26.1% who had positive results in those tested for cannabis and methamphetamines, respectively. Patients admitted to the MHU spend approximately two to three days in the emergency unit before being transferred to the unit, and this could explain the lower positive rates of methamphetamine tests compared to cannabis tests.

Of the 95 participants who used substances, the majority were male. Furthermore, being a male was strongly associated with substance use (*p* < 0.001), specifically cannabis use (*p* < 0.001) and methamphetamine use (*p* = 0.04). Other studies have also shown a higher proportion of male patients among those who use substances in first-episode psychosis.^35,36,37,38^

Statistics for EC drug treatment centres show that alcohol is a commonly used substance (45.2%) followed by cannabis (17.6%) and methamphetamine (16.2%), with average ages of 41 years for alcohol, 20 years for cannabis, and 24 years for metamphetamine.^39^ The difference in the prevalence of substance use in EC drug treatment centres compared to the findings of the current study could be because of the older age of treatment-seeking individuals in other centres compared to our study setting. The EC treatment centre figures were also drawn from an outpatient sample who did not necessarily have any comorbid psychiatric disorder.

In this study, substance use was not significantly associated with ethnicity or with level of education. Previous studies have shown similar results,^16^ while others have shown that those with substance use, especially cannabis use, have poor academic progress.^26^ Unemployment was associated with a higher prevalence of substance use (*p* < 0.001) in this study. The impact of substance use on employment remains unclear with some studies showing negative impact,^12,40^ while others show a complete absence of impact.^41,42^

The limitations associated with retrospective chart reviews are well known. This study was limited because of non-standardisation of assessing doctors who might have had varied clinical experience (intern doctors compared to specialist psychiatrists). Data collection was dependent on proper documentation of clinical notes and, as a result, there were missing data that could not be recovered. Additional data on the frequency of use, amount used and age of onset of drug use were not available in this sample.

## Conclusion

In keeping with national and international literature, this study showed a high prevalence of substance use in patients with FEP. The most commonly used substances were cannabis, alcohol and stimulants. The study findings highlight the need for mental health services in the EC to focus on dual diagnosis in order to address the challenge of substance abuse and its association with FEP. With the recent constitutional ruling on personal use of cannabis in South Africa, the high prevalence of use and its clinical correlates in association with first-episode psychosis requires further monitoring and evaluation to detect any changes in trends which may affect utilisation of services.^43^ Preventative strategies focusing on substance use disorder could also assist in addressing the growing burden of mental disorders in this region. Further prospective research is needed to confirm the higher prevalence of substance use reflected in this study.
